# Dizziness in peri- and postmenopausal women is associated with anxiety: a cross-sectional study

**DOI:** 10.1186/s13030-018-0140-1

**Published:** 2018-12-12

**Authors:** Masakazu Terauchi, Tamami Odai, Asuka Hirose, Kiyoko Kato, Mihoko Akiyoshi, Mikako Masuda, Reiko Tsunoda, Hiroaki Fushiki, Naoyuki Miyasaka

**Affiliations:** 1 Department of Women’s Health, Tokyo Medical and Dental University, Yushima 1-5-45, Bunkyo, Tokyo, 113–8510 Japan; 2 Department of Obstetrics and Gynecology, Tokyo Medical and Dental University, Yushima 1-5-45, Bunkyo, Tokyo, 113–8510 Japan; 3 Department of Speech, Language, and Hearing Therapy, Mejiro University, Ukiya 320, Iwatsuki, Saitama, 339-8501 Japan

**Keywords:** Climacterium, Menopause, Vertigo, Anxiety disorders, Quality of life, Dizziness

## Abstract

**Background:**

Although dizziness is one of the most common symptoms of menopause, the underlying mechanism is not precisely known. Therefore, this study aimed to investigate the prevalence of, and the factors associated with, dizziness in peri- and postmenopausal women.

**Methods:**

We conducted a cross-sectional study in which we analyzed the first-visit records of 471 Japanese women aged 40 to 65 years who enrolled in a health and nutrition education program at a menopause clinic. The prevalence of dizziness was estimated according to the participants’ responses to the Menopausal Health-Related Quality of Life Questionnaire. The background characteristics of age, menopause status, body composition, cardiovascular parameters, basal metabolism, and physical fitness; other menopausal symptoms, including vasomotor, insomnia, depression, and anxiety symptoms; and lifestyle characteristics were assessed for their associations with dizziness.

**Results:**

The percentage of women who suffered from dizziness once a week or more frequently was 35.7%. Compared to the women without dizziness, those with the symptom were younger; had a higher body weight, body mass index, body fat percentage, muscle mass, and waist-to-hip ratio; had higher systolic pressure; were slower in reaction time; had higher physical and psychological symptom scores of menopause; exercised less regularly; and consumed less alcohol. A multivariate logistic regression analysis revealed that the anxiety symptom, which was evaluated by the Hospital Anxiety and Depression Scale, was the sole factor that was independently associated with dizziness (adjusted odds ratio 1.14; 95% confidence interval 1.08–1.20).

**Conclusions:**

Dizziness is highly prevalent in Japanese peri- and postmenopausal women and it is associated with anxiety. The treatment of anxiety in this population might improve the symptom.

## Background

Women who are in the menopausal transition and postmenopausal periods are affected by a variety of symptoms, such as hot flashes, night sweats, vaginal dryness, depression, anxiety, and insomnia. Non-specific somatic symptoms are also common, including muscle and joint pain, tiredness, and dizziness. Dizziness, which is “an imprecise term which may refer to a sense of spatial disorientation, motion of the environment, or lightheadedness,” according to the Medical Subject Headings of the United States National Library of Medicine [1], is often used interchangeably for vertigo, presyncope, disequilibrium, or lightheadedness. The symptom is itemized in most menopausal symptom inventories, such as “feeling dizzy or faint” in the Greene Climacteric Scale [2], or “dizzy spells” in the Women’s Health Questionnaire [3]. There may be some ethnic differences in the prevalence of the symptom in middle-aged women, as shown in a study by Anderson et al., in which 25.1% of Australian local residents aged 45–60 years reported “feeling dizzy or faint” at least a little, while 41.8% of their Japanese counterparts suffered from the symptom (*p* < 0.001, chi-squared test) [4].

However, despite its prevalence, the precise mechanisms underlying dizziness in middle-aged women have yet to be elucidated, though some hypotheses have been proposed. For example, Yang et al. recently reported that ovariectomized mice have significant balance behavioral deficits probably because the estrogen deficiency compromises otoconia maintenance and anchoring by reducing the expression of the otoconial component and anchoring proteins [5]. Therefore, in the present study, we sought to determine the prevalence of dizziness in Japanese middle-aged women who were attending a menopause clinic and to investigate the factors associated with the symptom in this population.

## Methods

### Patient recruitment

In this cross-sectional study, we retrospectively analyzed the first-visit records of 471 Japanese peri- and postmenopausal women aged 40–65 years who had been enrolled in the Systematic Health and Nutrition Education Program at the menopause clinic of the Tokyo Medical and Dental University Hospital from November 2007 to June 2016. All women enrolled in this program were referred to our clinic for the treatment of menopausal symptoms. Prior to beginning the study, the research protocol was approved by the Tokyo Medical and Dental University’s Review Board (approval number 774), and informed consent was obtained from all participants. All study procedures were performed in accordance with the Declaration of Helsinki.

### Menopause definitions

Women were defined as premenopausal if they had experienced regular menstrual cycles in the past 3 months, as perimenopausal if they had had a menstrual period within the past 12 months but had a missed period or had an irregular cycle in the past 3 months, as postmenopausal if they had no menstrual period in the past 12 months, and as surgically induced menopause if they had had a hysterectomy.

### Questionnaires

Data regarding the menopausal symptoms and quality of life during the past month were collected using the Menopausal Health-Related Quality of Life Questionnaire (MHR-QOL) and the Hospital Anxiety and Depression Scale (HADS). These data were collected by the physicians and nutritionists who interviewed the women during their initial visit.

The MHR-QOL, which was developed and validated in our clinic [6–18], is a modification of the Women’s Health Questionnaire [3, 19]. It contains 38 items, which are scored on a four-point or binary scale and cover four major domains of health during menopause (physical health, mental health, life satisfaction, and social involvement). The item scores were used to indicate the symptom frequency (0–1 times per month = 0; 1–2 times per week = 1; 3–4 times per week = 2; almost every day = 3). The physical health domain consists of nine items that assess vasomotor, somatic, and urinary symptoms. The average of the two vasomotor items (“hot flashes” and “night sweats”) was defined as the vasomotor symptom score. The mental health domain consists of 12 items that assess depressed mood, cognitive difficulty, anxiety and fear, sexual function, sleep problems, and low self-esteem. The average of the two sleep problem items (“difficulty initiating sleep” and “non-restorative sleep”) was defined as the insomnia symptom score.

The HADS was developed by Zigmond and Snaith [20] as a reliable instrument for screening clinically significant anxiety and depression in women visiting a general medical clinic, and the Japanese translation was performed by Kitamura [21]. The HADS has seven items (odd items) that comprise the anxiety subscale and another seven items (even items) that comprise the depression subscale. The women responded to these items using a four-point Likert scale. Women who had a score of 8–10 points were considered likely to be experiencing anxiety or depression, and those who had a score of 11–21 points were considered to be definitely experiencing anxiety or depression.

### Physical assessment

The height, weight, waist circumference, and hip circumference of the participants were measured to determine their body mass index (BMI) and waist-to-hip ratio. Their body composition, including body fat, muscle mass, water mass, and visceral fat level, was assessed using a bioimpedance analyzer (MC190-EM; Tanita, Tokyo, Japan). The cardiovascular parameters, including systolic blood pressure, diastolic blood pressure, heart rate, cardio-ankle vascular index (arterial stiffness), and ankle-brachial pressure index, were measured using a vascular screening system (VS-1000; Fukuda Denshi, Tokyo, Japan). Additionally, their resting energy expenditure was measured using a portable, indirect calorimeter (Metavine-N VMB-005 N; Vine, Tokyo, Japan).

Physical fitness was assessed with tests for power, reaction time, and flexibility. Hand-grip strength was measured twice for each hand with a hand dynamometer (Yagami, Nagoya, Japan), with the larger value from each hand used to calculate the average hand-grip strength (kgf). Reaction time was measured with the ruler drop test using a wooden ruler that was 60 cm in length and weighed 110 g (Yagami, Nagoya, Japan). Briefly, the tester held the ruler such that the 0 cm line was surrounded by, but not touching, the seated participant’s outstretched fingers and thumb. The tester then dropped the ruler at an arbitrary time and the participant attempted to catch it as quickly as possible, and the distance that the ruler fell before being caught was recorded. The test was repeated seven times. After omitting the largest and smallest values, the remaining five values were pooled to give an average reaction time (cm). The flexibility was measured using a sit and reach box (Yagami, Nagoya, Japan).

### Lifestyle characteristics

The following lifestyle characteristics were also assessed: the habit of regular exercise (yes, no); amount of daily caffeinated beverage consumption (more than 3 cups, 1–3 cups, none); frequency of alcohol consumption (daily, sometimes, never); and the habit of smoking (more than 20 cigarettes per day, 1–20 cigarettes per day, none).

### Factors associated with dizziness

Women were defined as experiencing dizziness if they scored 1, 2, or 3 on the second item in the MHR-QOL’s physical health domain, indicating that they suffered from the symptom once a week or more frequently. Women with and without dizziness were then compared for age, menopausal status, body composition, cardiovascular parameters, basal metabolism, physical fitness, physical and psychological symptoms of menopause (i.e., vasomotor, insomnia, depression, and anxiety symptom scores), and lifestyle characteristics. Next, the factors that significantly differed (*p* < 0.20) between these two groups at the univariate level were selected as the explanatory variables for a multivariate logistic regression analysis that was conducted to identify the factors that are independently associated with the response variable of dizziness using a stepwise variable selection procedure (*p* < 0.05).

### Statistical analysis

The group comparison was performed using the unpaired *t*-test, Mann Whitney U-test, or Fisher’s exact test. The variables that significantly differed between the groups were tested for multicollinearity using Pearson’s or Spearman’s correlation analyses. All statistical analyses, including the multivariate logistic regression analysis detailed above, were performed with SAS version 9.2 (SAS Institute, Cary, NC, USA).

## Results

The average age of the participants was 51.2 ± 5.0 years (mean ± standard deviation). The rates (0–1 times per month, 1–2 times per week, 3–4 times per week, and almost every day) of dizziness among the 471 women screened are shown in Table 1. The percentage of women who complained of dizziness once a week or more frequently was 35.7%.

**Table 1:** Percentages of women with dizziness in our study population (*n* = 471)

How often the women experienced dizziness	Number	Percent
0–1 time a month (none)	303	64.3
1–2 times a week (mild)	106	22.5
3–4 times a week (moderate)	29	6.2
Almost every day (severe)	33	7.0

The variables that significantly differed (*p* < 0.20) at the univariate level between the women with (*n* = 168) and without (*n* = 303) dizziness were as follows: age (y); body weight (kg); BMI (kg/cm^2^); body fat (%); muscle mass (kg); waist-to-hip ratio (%); systolic pressure (mmHg); reaction time (cm); the vasomotor, insomnia, depression, and anxiety symptom scores; the habit of regular exercise; and alcohol consumption. Compared to the women without dizziness, those with the symptom were younger; had a higher body weight, BMI, body fat percentage, muscle mass, and waist-to-hip ratio; had higher systolic pressure; were slower in reaction time; had higher physical and psychological symptom scores of menopause; exercised less regularly; and consumed less alcohol (Table 2).

**Table 2:** Comparison of the background characteristics between the women with and without dizziness

	Not suffered from dizziness (*n* = 303)	Suffered from dizziness (*n* = 168)	*p*-value
*Age and menopause status*			
Age, years, mean (*SD*)	51.4 (5.1)	50.7 (4.7)	0.106^a^
Menopausal status (premenopausal /perimenopausal/postmenopausal/surgical menopause, %)	26.1/16.2/43.6/14.2	28.0/22.6/35.7/13.7	0.238^c^
*Body composition, mean (SD)*			
Height, cm	157.0 (5.3)	157.4 (5.7)	0.500^a^
Body weight, kg	52.7 (8.8)	54.4 (9.6)	0.049^a^
Body mass index, kg/m^2^	21.4 (3.4)	22.0 (3.8)	0.067^a^
Body fat, %	26.8 (7.5)	27.9 (7.9)	0.127^a^
Muscle mass, kg	35.9 (2.9)	36.3 (3.1)	0.147^a^
Water mass, kg	27.4 (4.0)	27.8 (3.2)	0.328^a^
Waist-to-hip ratio, %	85.9 (6.0)	86.8 (7.0)	0.121^a^
Visceral fat level	4.7 (2.5)	5.0 (2.8)	0.215^b^
*Cardiovascular parameters, mean (SD)*			
Systolic pressure, mmHg	124.8 (15.9)	127.6 (17.9)	0.083^a^
Diastolic pressure, mmHg	79.9 (11.0)	80.6 (12.4)	0.507^a^
Heart rate, beats/min	64.0 (11.7)	63.3 (9.8)	0.494^a^
Cardio-ankle vascular index	7.52 (0.78)	7.48 (0.75)	0.610^a^
Ankle-brachial pressure index	1.11 (0.06)	1.11 (0.06)	0.334^a^
*Basal metabolism, mean (SD)*			
Body temperature, (°C)	36.3 (0.48)	36.3 (0.45)	0.757^a^
Resting energy expenditure, kcal/day	1591 (387)	1625 (430)	0.385^a^
*Physical fitness, mean (SD)*			
Hand-grip strength, kgf	25.2 (4.6)	25.0 (4.9)	0.569^a^
Reaction time, cm	21.8 (4.1)	22.6 (4.3)	0.053^a^
Sit and reach test, cm	36.5 (9.7)	37.2 (9.4)	0.489^a^
*Physical symptoms, mean (SD)*			
MHR-QOL vasomotor symptom score	1.7 (2.0)	2.4 (2.1)	< 0.001^b^
*Psychological symptoms, mean (SD)*			
MHR-QOL insomnia symptom score	2.0 (2.2)	2.7 (2.1)	< 0.001^b^
Hospital Anxiety and Depression Scale’s depression subscale score	6.2 (4.0)	8.1 (3.9)	< 0.001^b^
Hospital Anxiety and Depression Scale’s anxiety subscale score	6.9 (3.9)	8.8 (3.6)	< 0.001^b^
*Lifestyle characteristics, %*			
Habit of regular exercise, yes/no	47.2/52.8	34.3/65.7	0.008^c^
Amount of daily caffeinated beverage consumption, more than 3 cups/1–3 cups/none	66.6/24.8/8.6	61.3/32.1/6.5	0.208^c^
Frequency of alcohol consumption, daily/sometimes/never	11.6/31.6/56.8	7.1/28.6/64.3	0.174^c^
Habit of smoking, more than 20 cigarettes per day/1–20 cigarettes per day/none	2.3/8.3/89.4	3.6/8.3/88.1	0.728^c^

*MHR-QOL* Menopausal Health-Related Quality of Life Questionnaire

^a^Unpaired *t*-test

^b^Mann Whitney U-test

^c^Fisher’s exact test

Of these variables, body weight and body fat had significant multicollinearity with the BMI (Pearson’s *r*/*p*: 0.906/< 0.05 and 0.917/< 0.05, respectively), and they were excluded from the following multivariate logistic regression analysis. Of the remaining factors, the HADS’ anxiety score was revealed to be the sole factor that was independently associated with dizziness (adjusted odds ratio 1.14; 95% confidence interval 1.08–1.20) (Table 3).

**Table 3:** Factors independently associated with dizziness

	Adjusted odds ratio (95% confidence interval)	*p*-value
Hospital Anxiety and Depression Scale’s anxiety subscale score	1.14 (1.08–1.20)	*p* < 0.001

A multivariate logistic regression analysis using a stepwise variable selection procedure was performed to identify the independent risk factors.

## Discussion

In our analysis of 471 middle-aged women, more than one third suffered from dizziness once a week or more frequently, and the sole factor that was independently associated with the symptom was revealed to be anxiety. The percentage of women who experienced dizziness in our study participants, who were aged 40–65 years and recruited in a clinical setting (35.7%), is close to that observed in the Japanese local residents aged 45–60 years in Anderson’s study (41.8%), which was significantly higher than that in Australian local residents in the same study (25.1%) [4]. This gap in the prevalence of dizziness in middle-aged women could be attributable to ethnic differences in the biological mechanisms underlying the symptoms, or to ethnic differences in the somatization of anxiety, considering that the association between dizziness and anxiety was confirmed in the current study. Interestingly, when Hoge et al. compared the somatic and psychological symptoms of Nepali and American patients diagnosed with generalized anxiety disorder, they found that the somatic symptom scores, including that of dizziness, were significantly higher in the former, while the psychological symptom scores were higher in the latter, possibly due to the differences in the cultural traditions of describing emotional distress [22].

Dizziness and anxiety have long been known to coexist, and a bidirectional relationship has been presumed in terms of dizziness-evoked anxiety (a somatopsychic model) and anxiety-evoked dizziness (a psychosomatic model) [23]. A number of hypotheses have been proposed to explain the mechanisms underlying this interplay based on the findings obtained from animal studies. For example, in rats subjected to hypergravity generated by centrifugation, the vestibular stimuli activated the central nucleus of the amygdala, which is a crucial locus for retaining the memory of stimuli for conditioned fear and taste-aversion learning, as determined by the induction of the Fos expression [24]. On the other hand, guinea pigs chronically administered with vasopressin, which is a small neuropeptide produced by the hypothalamic neurons that respond to stressful environmental challenges, developed endolymphatic hydrops (as defined by the increase in the scala media area in the mid-modiolar sections of the cochlea), which mimics the pathology of Meniere’s disease [25].

Furthermore, the concept of “psychiatric dizziness” is supported by the clinical observations that some dizziness occurs exclusively in combination with other symptoms as part of a recognized psychiatric symptom cluster and that this symptom cluster is not itself related to vestibular dysfunction [26]. It is also supported by the finding that psychotherapy, such as cognitive behavioral therapy combined with relaxation techniques with or without vestibular rehabilitation, is effective for improving dizziness to some extent [27]. The finding of the current study that the anxiety symptom score was the sole factor that was independently associated with dizziness in peri- and postmenopausal women highlights that the association between these two symptoms also occurs in this specific population.

Although the multiple logistic regression analysis eliminated psychological symptoms of menopause other than anxiety as factors independently associated with dizziness, depression is also an established contributor to the symptom. Horii et al. revealed that a selective serotonin reuptake inhibitor fluvoxamine improved dizziness in male and female patients whose HADS score decreased with the treatment, suggesting that some of the symptoms were induced by depression [28]. In the same study, HADS-anxiety subscale and depression subscale showed a high correlation both in pre- and post-treatment periods, suggesting that anxiety and depression symptoms were closely related with each other in the patients suffering from dizziness. Our finding that anxiety is the only factor independently associated with dizziness implies that the anxiety was slightly more interconnected with dizziness than depression in the group of women with the symptom in our current study population.

The fact that dizziness is one of the symptoms that middle-aged women most frequently complain about, combined with the report from a hospital survey in Omaha, Nebraska that found that the estimated prevalence (per 100,000 local residents) of benign paroxysmal positional vertigo (BPPV) in women sharply rose from 77.2 in their thirties to 149.7 in their forties and kept increasing to the level of 263.5 in their seventies (compared to that in men, which was 38.2 in their thirties, 47.5 in their forties, and 181.0 (11.4%) [29]), implies that estrogen fluctuation and deficiency in peri- and postmenopausal women might play a role in the pathogenesis of BPPV, as well as a wider spectrum of dizziness. A recent animal study supports this hypothesis as it revealed that ovariectomized mice have significant balance behavioral deficits and that estrogen deficiency compromises otoconia maintenance and anchoring by reducing the expression of otoconial component and anchoring proteins, in addition to causing ectopic debris formation in the ampulla due to aberrant matrix protein expression [5]. More clinically, a placebo-controlled randomized controlled trial in Sweden demonstrated that hormone therapy significantly decreased sway velocity in elderly women who had low levels of baseline serum estradiol [30], which is in line with a cross-sectional analysis in Brazil that showed that hormone therapy users had significantly smaller oscillation than non-users, independent of the muscle strength in their lower limbs [31].

However, the ratio of women with the status of premenopausal/perimenopausal/postmenopausal/surgical menopause was not significantly different in the groups with and without dizziness in our current analysis (Table 2), which suggests that the subjective complaint of dizziness in this population was not affected by the sufficiency, deficiency, or fluctuation of estradiol. The discrepancy could be explained by a difference between the mechanisms underlying BPPV or impaired postural balance and the general dizziness experienced by peri- and postmenopausal women.

The present study was limited by the relatively narrow study population, which included only middle-aged women attending our menopause clinic. It is therefore difficult to extrapolate our results to a wider population. The history of the dizziness was not taken in detail, and the vestibular function of the participants was not evaluated. Furthermore, the cross-sectional design of this study prevented the determination of a causal relationship between the dizziness and anxiety.

## Conclusions

Dizziness is highly prevalent in Japanese peri- and postmenopausal women and it is associated with anxiety. The treatment of anxiety in this population might improve the symptom.

## Abbreviations

BMI: body mass index
BPPV: benign paroxysmal positional vertigo
HADS: Hospital Anxiety and Depression Scale
MHR-QOL: Menopausal Health-Related Quality of Life Questionnaire
